# Real-Time Multi-Scale Face Detector on Embedded Devices

**DOI:** 10.3390/s19092158

**Published:** 2019-05-09

**Authors:** Xu Zhao, Xiaoqing Liang, Chaoyang Zhao, Ming Tang, Jinqiao Wang

**Affiliations:** 1 National Laboratory of Pattern Recognition, Institute of Automation, Chinese Academy of Sciences, Beijing 100190, China; xu.zhao@nlpr.ia.ac.cn (X.Z.); xiaoqing.liang@nlpr.ia.ac.cn (X.L.); tangm@nlpr.ia.ac.cn (M.T.); jqwang@nlpr.ia.ac.cn (J.W.); 2 University of Chinese Academy of Sciences, Beijing 100049, China

**Keywords:** face detection, ARM-based devices, model acceleration, computer vision

## Abstract

Face detection is the basic step in video face analysis and has been studied for many years. However, achieving real-time performance on computation-resource-limited embedded devices still remains an open challenge. To address this problem, in this paper we propose a face detector, EagleEye, which shows a good trade-off between high accuracy and fast speed on the popular embedded device with low computation power (e.g., the Raspberry Pi 3b+). The EagleEye is designed to have low floating-point operations per second (FLOPS) as well as enough capacity, and its accuracy is further improved without adding too much FLOPS. Specifically, we design five strategies for building efficient face detectors with a good balance of accuracy and running speed. The first two strategies help to build a detector with low computation complexity and enough capacity. We use convolution factorization to change traditional convolutions into more sparse depth-wise convolutions to save computation costs and we use successive downsampling convolutions at the beginning of the face detection network. The latter three strategies significantly improve the accuracy of the light-weight detector without adding too much computation costs. We design an efficient context module to utilize context information to benefit the face detection. We also adopt information preserving activation function to increase the network capacity. Finally, we use focal loss to further improve the accuracy by handling the class imbalance problem better. Experiments show that the EagleEye outperforms the other face detectors with the same order of computation costs, on both runtime efficiency and accuracy.

## 1. Introduction

Face detection is a hot topic in computer vision. It is the basic step for face-related applications, such as face recognition, face attribute classification, face beautification, etc. In the last two decades, many approaches have been proposed to solve it [1,2,3,4,5,6,7,8,9,10,11,12,13]. The faces in the wild vary in scales and pose, and they usually appear in cluttered backgrounds. These situations increase the difficulty of the face detection.

The two main focuses of the face detection task are the speed and accuracy of the proposed approaches. They are both important. Usually, the approaches with high computational complexity perform better, but they run in a low running speed. Nowadays, most of the face detection applications are deployed on embedded devices. Embedded devices usually have low computation resources. Therefore, building efficient methods with a good balance of speed and accuracy is very important. Another bottleneck of the embedded devices is the limited amount of available system memory, which is discussed in [14,15]. Keeping the face detector’s memory complexity low is also important for embedded devices. Fortunately, modern embedded devices usually have large enough memory for high-speed face detectors, for example the Raspberry Pi 3b+, the RK3399, and most recent mobile phones. Therefore, this paper mainly focuses on designing effective strategies for building efficient face detectors with a good balance between speed and accuracy.

Traditional face detection methods usually follow the sliding-window fashion. Viola–Jones [16] is the pioneering method for face detection. It designs the Haar-like feature and uses the Adaboost algorithm to classify each window on the image pyramid. After that, many traditional methods are proposed. They focus on designing effective hand-craft features and building powerful classifiers. For example, ACF (Aggregated Channel Features) [17] uses the aggregated channel features and adopts the fast feature pyramid building strategy. These traditional methods could not deal with complex situations because of their weak-semantic features. Moreover, since they usually adopt the image pyramid to deal with the multiple face scales, they have limited advantage in running speed.

Currently, more and more deep learning-based methods are proposed. The state-of-the-art methods usually regard the faces as a special case of general objects, and most of them are inherited from general object detection methods. Some of them are based on faster RCNN (Region-based Convolutional Neural Networks) [18], they follow the two-stage face detection pipeline. In the first stage, they generate the candidate face proposals to filter out most of the background samples. Then, in the second stage, they get the final detection result by classifying the proposals into faces and non-faces, as well as further regressing them to the ground-truth location. Though there are some works to improve the running efficiency of two-stage methods, the two-stage methods are still not friendly with embedded devices.

On the other hand, most of the current state-of-the-art methods follow the single-stage framework. Methods like SFD (Single Shot Scale-Invariant Face Detector) [10], SSH (Single Stage Headless Face Detector) [5], PyramidBox [7], and FANet (Feature Agglomeration Networks) [9] are based on SSD (Single Shot Multi-box Detector) [19] detector. SFD [10] adopts the VGG-16-based [20] SSD detector. It proposes several guidelines about the anchor setting strategy to improve the recall rate of the face detector. SSH [5] removes the fully-connected layers of VGG-16 to speed up the running speed. SSH also designs a context module on the predicting layers to utilize context information. PyramidBox [7] designs multiple strategies to utilize the context information to improve the face detection results. FANet [9] designs a novel hierarchical feature pyramid to better merge the feature maps of different stages. Another category of methods are based on RetinaNet [21], like SRN (Selective Refinement Network) [2] and FAN (Face Attention Network) [22]. SRN [2] is inspired by the RefineDet [21]. It appends a refinement branch to refine the classification results for small objects and regression results for large objects. FAN [22] designs the attention module to improve the face detection performance in some hard cases, like occlusions and person wearing masks. These single-stage methods are generally faster than the two-stage methods. However, they tend to use large backbones (like VGG-16 or ResNet [23]) or design heavy predicting heads to guarantee the performance, which makes them less efficient on embedded devices.

There are only a few works aimed at designing highly efficient face detectors. MTCNN (Multi-Task Convolutional Neural Network) [24] designs the cascade convolutional neural networks to filter background patches in a coarse to fine way. MTCNN [24] is feasible to adapt different running speed requirements of different devices, but its performance is not satisfactory in dealing with complex situations. Faceboxes [25] is based on the SSD framework. They proposed two useful modules for building efficient networks. The modules are the rapidly digested convolutional layers (RDCL) and the multiple scale convolutional layers (MSCL). By designing an efficient face detection backbone based on the two modules, the Faceboxes detector is able to run real-time on the x86 CPU based desktop. However, it still cannot meet the real-time requirement for embedded devices.

Besides the above works that are specific to face detection, there are several works about building efficient classification networks. MobileNet [26] uses depth-wise convolutions and point-wise convolutions together to replace the regular convolutions. MobileNet-v2 [27] proposes the inverse residual blocks and linear bottlenecks to improve the accuracy of the depth-wise convolutions based networks (i.e., MobileNet). ShuffleNet [28] converts the point-wise convolutions in MobileNet to group point-wise convolutions and uses the channel-shuffle operation to exchange information between channels. ShuffleNet-v2 is the improved version of ShuffleNet. ShuffleNet-v2 [29] find that beyond minimizing the FLOPS, four practical guidelines should be considered for building efficient networks that run fast on real devices. These networks are friendly with embedded devices, but they are mainly designed for classification tasks. Although they can be directly applied in detection tasks, we think that several modifications on them should be considered to build more efficient networks for detecting faces.

An efficient face detection network should achieve a good trade-off between speed and accuracy. To build such an efficient network, we think there are two considerations. On one hand, the architecture of the network should be efficient while it should maintain the necessary network capacity for being accuracy. On the other hand, different from the general strategies proposed in recent face detectors that improve the accuracy at the expense of reducing the speed a lot [2,7], the strategies to improve the efficient network’s accuracy should bring less additional computation costs as possible. In this paper, we use the floating-point operations per second (FLOPS) as the index of the detector’s speed, for it reflects the computation complexity of the detection network and it is not affected by specific devices and specific inference libraries. Therefore, the key is to build networks with low FLOPS and enough capacity and propose strategies to improve its accuracy without adding too many FLOPS.

**Figure 1:**
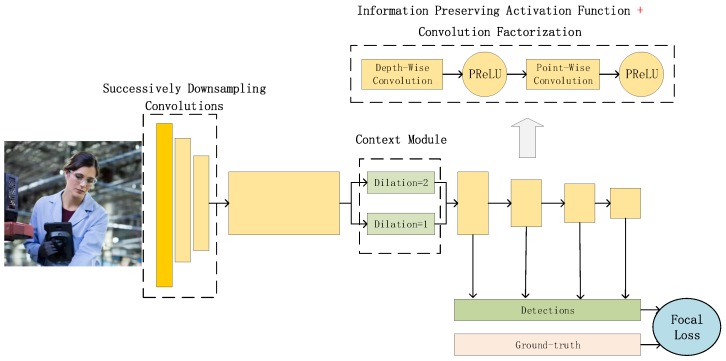
The overview of the network architecture of EagleEye face detector. The detection network is built using the information preserving activation function and the convolution factorization in almost all the backbone layers and the predicting layers.

In this paper, to solve the problem that current face detectors could not reach a good balance of speed and accuracy on embedded devices, we propose an arm based embedded devices oriented face detector, EagleEye. The overview architecture of EagleEye is shown in Figure 1. EagleEye is inspired by many of the above works. We first propose two strategies to reduce the computation cost without reducing the accuracy too much. Then we propose three strategies to improve the accuracy without adding too much computation cost. To reduce the FLOPS without sacrificing too much capacity, firstly, we adopt the convolution factorization module to use the depth-wise convolutions and point-wise convolutions to build the whole detector as efficient as possible; secondly, we set the successive downsampling convolutions in the several beginning stages of the network which remove the unnecessary layers. To improve the accuracy without adding too much computational cost, firstly, we design an efficient multi-scale context module to utilize context information to improve the detection accuracy; secondly, we use the information-preserving activation function to increase the capacity of the network; thirdly, we introduce the focal loss to help the light-weight network to deal with the class-imbalance problem during the training process. We build the EagleEye face detector with the above four strategies. It achieves 50 ms per frame on an ARM A53 based embedded device, the Raspberry Pi 3b+. It also achieves 96.1 mAP on FDDB dataset.

The contributions of this paper are two-fold. Firstly, we propose five guidelines for building efficient face detectors. Secondly, we design the EagleEye face detector, which achieves good performance on ARM devices.

## 2. Related Work

Our EagleEye detector is inspired by many previous works. MobileNet [26], MobileNet-v2 [27], Xception [30], ShuffleNet [28], and ShuffleNet-v2 [29] also adopts the depthwise convolution for efficient running on devices, but they are created as classification networks. We adopt the depth-wise convolution to build more efficient networks and extend it to dilated depth-wise convolutions to extract context information with little computation costs.

The Faceboxes [25] use a rapidly digested convolutional layers (RDCL) module to quickly reduce the resolution of feature maps. This is similar to our successive downsampling convolutions module. However, there are several differences between them. Firstly, EagleEye does not have the pooling layer for it would decrease the accuracy of small faces. Secondly, we do not adopt the C.ReLu layer for its limited improvements as shown in Faceboxes. Thirdly, we do not use the large kernel sizes of seven or five in building EagleEye, for they are usually ignored by many the implement of deep learning inference libraries and they would increase the number of parameters of the detector.

The dilation convolution is widely used in semantic segmentation to increase the scale of the receptive field and introduce more context information, like the ASPP (Atrous Spatial Pyramid Pooling) in DeepLab [31] method. In object detection tasks, RFBNet (Receptive Field Block Networks) [32] use multiple dilation convolutions at each of the predicting branches. However, we think that adding a multiple-dilation-convolution module at each of the predicting branches is not efficient enough, so we propose to add it at the middle of the backbone to compute it only once. After that, the context information is continuously fed into the following layers. Using multiple dilation convolutions in the backbone as a context module is also proposed in ISSD (Improved Single Shot Object Detector) [33]. ISSD uses four split-dilatedConv-sum (dilatedConv means the dilated convolution operation) branches to extract multi-scale information. Because ISSD is for general object detection in scenes and EagleEye is for face detection, we remove the two branches with large dilation rates because only the human body regions are helpful in detecting faces. Moreover, we use the slice-dilatedConv-concat branches to reduce the input channels of each branch.

The non-saturated activation functions, like ReLU (Rectified Linear Unit), are the key to making deep convolutional neural networks outstanding. As discussed in [34], the non-saturated activation functions could solve the “exploding/vanishing gradient” problem and accelerate the convergence speed. Besides the most widely used method ReLU [35], its variants like leaky ReLU [36], PReLU (Parametric Rectified Linear Unit) [37], ELU (Exponential Rectified Linear Unit) [38], and Swish [39] are also proposed. The ReLU has the dead region below 0, thus it limited the capacity of the network. Many of these variants like ELU and Swish use the exponential function, which runs slowly on CPU. To improve the capacity of the network as well as keep the network efficient, the leaky ReLU and PReLU are suitable to be adopted to build the network. We find their effects are similar and choose the PReLU in EagleEye.

The class imbalance is a traditional problem in machine learning and object detection. It is usually alleviated by hard example mining [19,40] or re-weighting the loss values for different categories [41,42]. Lin et al. [43] proposed the Focal Loss for dealing with the class imbalance in one-stage detectors. In this paper, to achieve faster running speed, we design a one-stage face detector. In the proposed face detector, most of the anchors are in background regions. To make the gradient caused by different classes more balanced, we introduce the focal loss in training the light-weight face detector.

## 3. EagleEye

In this section, we give a detailed description of the proposed face detector, EagleEye, as shown in Figure 1. We use five key components for building it. Firstly, we adopt the depth-wise convolutions for building it. Secondly, we design a successive strided convolution layers module for downsampling the resolution of feature maps rapidly. Thirdly, we use dilated depth-wise convolutions for increasing the context information. Fourthly, we use the information preserving activation functions to increase the network’s capacity. Finally, we introduce the modified focal loss to improve the detector’s performance by handling the class imbalance better.

### 3.1. Baseline Detector

To better demonstrate the evolution of EagleEye, we firstly build a baseline backbone network.

Backbone. The baseline backbone network is built following the VGG-style, which is widely used in single stage face detection methods. Its architecture is shown in Table 1. It consists of seven stages. Like SSD and FPN, we predict the objects of different scales at multiple network layers with different depth and different strides. Specifically, we choose one layer from each of the stages four to seven as the predicting layers for four different face scales. These layers have the stride of 16, 32, 64, 128.

Multi-scale anchor Boxes. Following the strategy used in RPN (Region Proposal Networks) [44], SSD [19], and Faceboxes [25], we use predefined multi-scale anchor boxes to predict the faces with various scales. For each pixel on the feature maps of the predicting layer, we set two anchor boxes to it. By matching the ground-truth face boxes to the anchor boxes, each ground-truth face box would be matched with at least one anchor box. The scales of anchor boxes of each predicting layer are shown in the last column of Table 1. Moreover, since the faces tend to be a square shape, we set the anchors of the unified aspect ratio of 1:1.

As for the matching rules, we use the widely used IoU-based (IoU: Intersection over Union) matching rules. According to this rule, an anchor is matched to a ground-truth box if the IoU overlap between them is higher than a threshold. The definition of IoU is demonstrated in Equation (1).

(1) $$IoU\left(BOX_{1},BOX_{2}\right)=\frac{BOX_{1}\cap BOX_{2}}{BOX_{1}\cup BOX_{2}}.$$

According to many previous works, like YOLOv2 ( You Only Look Once, Version 2) [45] and S3FD (Single Shot Scale-Invariant Face Detector) [10], the average number of matched anchor boxes of each ground truth box should be high enough. This is the key to keep a high recall rate of detection results. Therefore, we set a relatively low matching threshold.

Predicting targets. In EagleEye, we use a 3×3 convolutional layers on the output feature maps of each predicting layer to generate a five-element vector (score,Δx,Δy,Δw,Δh) for each anchor which is assign to each one of its pixels. score∈[0,1] is the confidentce that the anchor ($x_{a},y_{a},w_{a},h_{a}$) is assigned to a face box. (Δx,Δy,Δw,Δh) is the offset between the anchor box with the ground-truth box. We could recover the detected bounding box ($x_{dt},y_{dt},w_{dt},h_{dt}$) by:

(2) $$\begin{array}{ccc} x_{dt} & = & x_{a}+w_{a}\times \Delta x,\\ y_{dt} & = & y_{a}+h_{a}\times \Delta y,\\ w_{dt} & = & w_{a}\times exp\left(\Delta w\right),\\ h_{dt} & = & h_{a}\times exp\left(\Delta h\right). \end{array}$$

After getting all predicted face bounding boxes, we use a standard greedy non-maximum suppression (NMS) algorithm to generate the final detection results.

Loss function. The output five-element vector (score,Δx,Δy,Δw,Δh) consists of two parts. The score is a classification task and the (Δx,Δy,Δw,Δh) is the regression task. For the classification task, we use the two-class cross entropy loss. Because the number of the positive samples is far lower than that of the negative samples, in the baseline detector, we use the online hard negative mining strategy to balance the ratio of them. For the regression task, we use the smooth-L1 loss for it is more robust.

Data augmentation. Data augmentation is widely used in single-stage object detection methods to improve their performance. In this paper, we use three kinds of augmentation methods to make the detectors fully trained.

- First, we randomly pad the sampled training images with 0 s to generate images with a larger size. Then use the randomly cropping method to crop the image patches and resize them to the unified size 512 × 512 as the training samples. When cropping, we make sure each cropped patch would have at least one face in it. This would augment the faces of various scales to make each predicting layer fully trained.
- Second, we randomly flip the images in the horizontal direction with a probability of 0.5 to generate new samples.
- Third, we distort the image in various color spaces. This could increase the robustness of the detector to illumination changes.

### 3.2. Convolution Factorization

Convolution factorization is the first strategy to reduce the computation complexity of the face detection network. The convolution factorization means that we factorize each standard convolution layer into a depth-wise convolution layer and a following point-wise convolution layer. The depth-wise convolution is firstly proposed in Xception [30], and is adopted as the core element of MobileNet [26]. The point-wise convolution is the regular 1 × 1 convolution.

**Figure 2:**
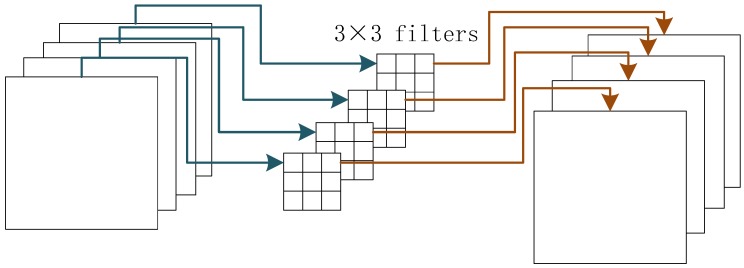
The illusion of the depth-wise convolution.

Depth-wise convolution. Figure 2 shows the computing process of the depth-wise convolution. The input feature map and the output feature map have the same number of channels. Each channel of the input feature map has a corresponding channel of the convolutional filters. Each filter only operates on one input channel. Comparing the standard convolutions that each filter operates on all input channels, the depth-wise convolution is very sparse, thus saving a lot of computation costs.

Point-wise Convolution. The point-wise convolution is the standard 1×1 convolution. It is used to aggregate the information among different channels. The standard convolution convolves the input feature map both in the spatial-wise and the channel-wise dimensions. The depth-wise convolution could convolve the input feature map in the spatial-wise dimension, but it loses the information exchange among the different channels. Therefore, depth-wise convolutions and point-wise convolutions are complementary to each other.

Effects of convolution factorization. By using convolution factorization, we factorize each standard convolution layer of the baseline detection network into a depth-wise convolution layer and a point-wise convolution layer. The convolution factorization has two advantages over directly adopting standard convolutions. Firstly, the parameters of the network becomes much less. We suppose the input channels is $N_{in}$ and the output channels is $N_{out}$, the regular 3×3 convolution has $3\times 3\times N_{in}\times N_{out}$ parameters. After convolution factorization, the parameters become $3\times 3\times N_{in}+1\times 1\times N_{in}\times N_{out}$ parameters. Secondly, the computation complexity is largely reduced. We use FLOPS as the index of computation complexity. We suppose the above convolution layer’s input and output feature maps’ spatial resolution are both H∗W. The FLOPS of regulation convolution would be $H\times W\times 3\times 3\times N_{in}\times N_{out}$, while after convolution factorization, the FLOPS would be $H\times W\times 3\times 3\times N_{in}+1\times 1\times N_{in}\times N_{out}$ × H × W.

Since the convolution factorization could reduce much computation costs, we adopt it in all convolutional layers of the face detection network except conv1, including the backbone and the predicting layers. The regular convolutions with the same number of input and output channels can easily be factorized into depth-wise and point-wise convolutions. For the convolution which has a different number of channels between its input and output feature maps, the channel number transformation is accomplished at the point-wise convolutions.

### 3.3. Successive Downsampling Convolutions

Successive downsampling convolutions is the second strategy to reduce the computation complexity of the face detection network. There are two key considerations when designing the network architectures, the network width, and network depth. Almost all base networks are like a pyramid. In other words, the resolutions of network layers are successively shrunk. The shrinking is usually done by a strided convolution layer or a pooling layer. We find that the pooling layers are not suitable for small objects, because they would lose much detailed information. Therefore, we do not use any pooling layer in building the backbone network of EagleEye.

The layers with the same output resolution are usually called a stage. In the popular network architectures, it is common that in each stage, each downsampling layer (stride=2) is followed by several stride–1 (stride=1) layers. This is to increase the depth of each stage. Each stage extracts features of different levels of semantic representation ability. By increasing the depth of a stage, the features it focuses on become finer. In this paper, since we are constrained by the low computation ability of embedded devices, we should keep the computation complexity of the backbone network low and reduce the number of the layers which come with fewer benefits. We think the stride–1 layers in the first several stages are less important because the features output by downsampling layers are already semantic strong enough to be input into their following stages. Therefore, we remove the stride–1 layers in the first two stages to reduce the computation costs. The third stage has only one stride–1 layer. We reserve it to prevent losing too much network capacity. Then we keep the stage depth of the following stages unchanged. The faces start to be predicted on stage 4, so stage 4’s features used for detecting faces should be semantic strong enough.

### 3.4. Context Module

**Figure 3:**
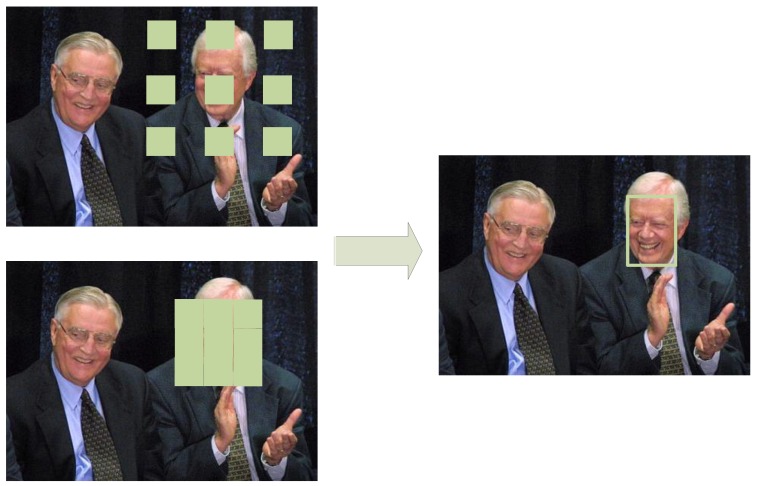
The illustration of using the head–shoulder region as the context information for face detection.

The context module is the first strategy to improve the detection accuracy without adding too much additional computation costs of the face detection network. To reduce the computation complexity of the designed network, we have to limit the capacity of the network. Therefore, the detection results may become inaccurate. To make up for the decrease of accuracy, we introduce the context information to help the network locate the faces. For example, the head–shoulder features are usually the indicator of the existing of faces above the bodies, as shown in Figure 3.

**Figure 4:**
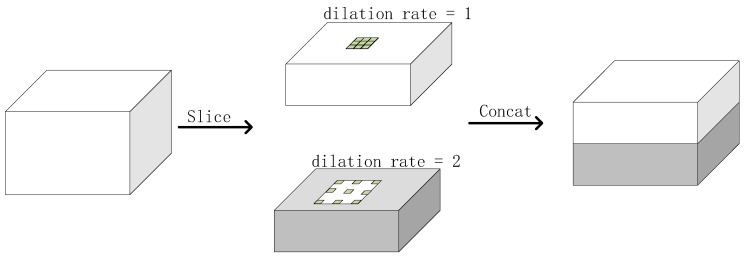
The illustration of the context module.

As discussed in [33], using the dilated convolution is a natural way to introduce context in the single stage detectors. Ref. [33] use four branches of convolutions with different dilation rates to extract multi-scale context information. Following [33], we design a multi-branch module to utilize multi-scale context information to improve detection performance. However, since our method is designed to run on embedded devices, we should not increase too much computation cost. Therefore, we made several modifications. Firstly, we use dilated depth-wise convolutions with different dilation rate for each branch. This largely reduces the computation complexity. Secondly, we use slice operation to equally divide the input feature map into two feature maps on the channel-wise dimension. This could reduce the number of channels of each branch. Another choice of the multi-branch module architecture is to make all branches share the same input feature map, as most of the modern networks do, like Inception, ResNet, and Faceboxes. Compared to the latter choice, the slice-branch-concat design leads to smaller inputs of each branch. Moreover, we do not set a large dilation rate for the dilation convolutions. Ref. [33] use the dilation rates of two, four, six, and eight to extract multi-scale contexts. However, unlike the general object detection tasks in [33] where the environment in the whole image could give clues for detecting the object, the faces rely less on the whole-image-level context. For example, the boats often appear on rivers, but the faces could appear at many scenes. Therefore, we limit the field of context regions by not using large dilation rates. The proposed context module in this paper is shown in Figure 4 and how the context module utilizes the head-shoulder region to help the face detection is shown in Figure 3.

### 3.5. Information Preserving Activation Function

The information preserving activation function is the second strategy to improve the detection accuracy without adding too much additional computation costs of the face detection network. It is an improvement on the ReLU activation function (Equation (3)), which is usually regarded to lose some information because of its dead region of (−∞, 0]. Because the face detection network with low computational cost has limited capacity, we could increase its capacity by reducing the information loss caused by the ReLU activation function. Therefore, we propose to replace the ReLU activation function with Leaky ReLU [36] or PReLU [37] in the baseline network. The leaky ReLU is demonstrated in Equation (4) and PReLU is demonstrated in Equation (5). Note the λ in Equation (4) is a constant value while the *a* in Equation (5) is the learnable parameter. The *a* is a vector whose length is the same as the number of the channels of its input feature maps.

(3) $$\mathrm{ReLU}\left(x\right)=\left\{\begin{array}{cc} x & \mathrm{if}\;x\geq 0,\\ 0 & \mathrm{if}\;x<0. \end{array}\right.$$

(4) $$\mathrm{LeakyReLU}\left(x\right)=\left\{\begin{array}{cc} x & \mathrm{if}\;x\geq 0,\\ \lambda x & \mathrm{if}\;x<0. \end{array}\right.$$

(5) $$\mathrm{PReLU}\left(x_{i}\right)=\left\{\begin{array}{cc} x_{i} & \mathrm{if}\;x_{i}\geq 0,\\ a_{i}x_{i} & \mathrm{if}\;x_{i}<0. \end{array}\right.$$

In experiments, we set the λ in Equation (4) to a fixed small value to 0.01. The leaky ReLu increases little additional computation costs. The increased FLOPS is less than the convolutional layers in order of magnitude. Therefore we note it is a good choice for improving the capacity of the light-weight networks.

### 3.6. Focal Loss

Focal loss is the third strategy to improve the detection accuracy. It is used in the training process and does not add any additional computation costs in the inference process. It improves the detector’s performance by dealing with the class imbalance problem in the training process better. Though the hard negative mining method used in the baseline can solve the class imbalance problem to some extent. The hard negative mining is still sub-optimal since it is hand-craft and discard all not-highest samples without considering whether these samples have high loss values or not. Suppose the output positive probability of sample *t* is $p_{t}$, the Focal Loss is shown in Equation (6). In Equation (6), α∈[0,1] is the weight for the losses of positive samples and γ∈[0,+inf) is the scaling factor for downsampling the easy samples. The optimal setting of α and γ in [21] is 0.25 and 2. In the embedded system based face detection task, the extremely hard examples such as heavy occlusions are less important. Therefore, in this paper, we change γ to 1 to avoid too much attention to the extremely hard examples.

(6) $$FL\left(p_{t}\right)=\left\{\begin{array}{cc} -\alpha {\left(1-p_{t}\right)}^{\gamma }\log\left(p_{t}\right) & \mathrm{if}\;\mathrm{the}\;\mathrm{sample}\;t\;\mathrm{is}\;\mathrm{positive},\\ -\left(1-\alpha \right){\left(p_{t}\right)}^{\gamma }\log\left(1-p_{t}\right) & \mathrm{if}\;\mathrm{the}\;\mathrm{sample}\;t\;\mathrm{is}\;\mathrm{negative}. \end{array}\right.$$

With the above strategies, we build the EagleEye based on the baseline detector, as shown in Figure 1 and Table 2.

## 4. Experiment

In this section, we firstly do sufficient ablation studies to demonstrate the effect of each strategy on the face detector’s speed and accuracy. Then we evaluate EagleEye on several common face detection datasets.

### 4.1. Experimental Details

Our face detection method was trained with the CAFFE [46] framework and tested with the NCNN (https://github.com/Tencent/ncnn) inference framework. NCNN is a high-performance neural network inference framework optimized for the mobile platform. It is friendly with the ARM processor based embedded devices. Equipped with the high-performance inference framework, the proposed efficient face detection method could run real-time on the embedded devices with low computation power.

We trained our face detection methods on 4 NVIDIA Titan X GPUs, with a batch size of 64 (=16 × 4) for 50,000 iterations with the learning rate starting from 0.02 and multiplying 0.1 at the 30,000 iteration. All network parameters are randomly initialized with the Xavier method. To test the efficacy of the proposed face detector, we use the Raspberry Pi1 3b+ which is an embedded device with very low computation power. Its CPU processor is the ARM A53 processor which is of 1.4 GHz.

### 4.2. Ablation Study

The wider face dataset [47] is a large face detection dataset. It has 32,203 images with 393,703 annotated faces, varying largely in scales, poses, occlusions, and illuminations. The images are divided into three splits, including 40% for training, 10% for validation, and 50% for testing. The faces are classified into three subsets according to their levels of detection difficult: easy, medium, and hard. Generally, the hard subset contains a great number of tiny faces. The official evaluation metric is the average precision (AP) for each subset. Since the current embedded devices based face detection task pays little attention to the small faces and extremely hard faces, we only choose the AP for the easy subset as the evaluation metric for the detector’s accuracy. For the evaluation metric of running speed, we choose the widely used metric: FLOPS, since it reflects the computation complexity of the networks and is not affected by specific devices and specific inference frameworks. In this section, we report the FLOPS of the models on VGA (Video Graphics Array) resolution inputs (i.e. 640 × 480).

In Table 3, we show the effectiveness of each strategy. The detectors are tested at the original resolution of each image. Firstly, the baseline designed in Section 3.1 reached 87.9 mAP. However, its FLOPS reached 440.3 M. On the NCNN based implementation, its running speed was 296 ms per image in VGA resolution, which is far from the real-time requirement. By convolution factorization, with a decrease of 4.2% mAP (easy), the FLOPS were largely reduced to 87.5 M (around 80% faster). After we adopted the successive downsampling module, the FLOPS were further reduced to 72.6 M and the mAP (easy) slightly dropped by 1.5%. The FLOPS of the light-weight detector were far lower than that of Faceboxes, which was 1796 M. Then we added the three strategies which aimed to improve the performance of the light-weight detector. The context module could improve 0.7% mAP (easy) without any increase on FLOPS. Moreover, when replacing the ReLU operation with information preserving activation function, PReLU, the mAP (easy) was raised by 0.4%. Finally, the focal loss improved the mAP (easy) to 84.1%. Thus Table 3 shows the convolution factorization and successive downsampling convolutions reduced the computation costs largely with sacrificing the accuracy by 5.7%, resulting in a light-weight detector. However, the following three strategies improved the accuracy of the light-weight detector by about 2%, making the light-weight detector more accurate.

In Table 4, we compare the result of using different activation functions. All three methods did not use the focal loss. We also kept other hyper-parameters’ setting the same among them. Generally, PReLU was better than the other two activation functions. Therefore we choose to use PReLU to build the EagleEye.

From Table 3, it can be seen that with the same settings about the hyper-parameters, the performance of EagleEye was still less than the baseline, but the FLOPS of EagleEye was far less than the baseline. This is reasonable because the models with fewer computation costs are usually less accurate than the complex one. To demonstrate the superiority of the architecture of EagleEye, we built a detector with roughly the same FLOPS with EagleEye by cutting 3/4 of the layer channels of the baseline, which is shown in Table 5 as $\frac{1}{4}$ Baseline. It can be seen that the EagleEye outperformed its counterpart method, $\frac{1}{4}$ baseline by a very large margin. This shows that the five strategies in Table 3 are important in designing light-weight networks. Firstly, the first two strategies can reduce the FLOPS while keeping the accuracy from dropping too large. Secondly, the latter three strategies can improve the performance of the detector, while keeping its low computation costs.

### 4.3. Runtime Efficiency

Following the common practice, we reported the runtime speed of EagleEye on the FDDB dataset [48] and compared it with the other methods. Since the previous methods all report their speed on desktop computers with Intel CPUs, we first tested the EagleEye using CAFFE library on Intel CPUs to directly compare their speeds. Besides, we tested the speed of two state-of-the-art methods: Faceboxes and MTCNN, on Raspberry Pi 3b+, an ARM Cortex-A53 based embedded device, by reimplementing them using NCNN library. Moreover, we also created another detector by simply replacing the backbone VGG network of the original SSD with 1/8 MobileNet network, to further show the effectiveness of our strategies. We reported these methods’ frames per second (FPS) on both devices. The results are shown in Table 6. It can be seen that the EagleEye ran fastest on both devices, especially on the ARM Cortex-A53 based embedded device. We note although the FLOPS of EagleEye was far lower than Faceboxes (75.3 M vs. 1796 M, on VGA resolution), fully exploiting its advantage needs proper implementation in the inference library. With proper optimization of the depth-wise convolutional layer, the EagleEye has the potential to have better runtime efficiency.

**Figure 5:**
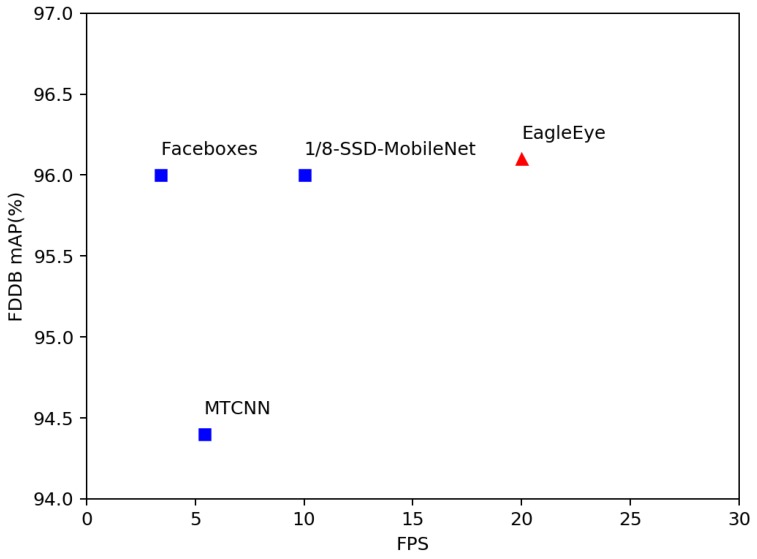
Speed (frames per second (FPS)) versus accuracy (mAP) on FDDB dataset. The speed (FPS) is tested on the ARM Cortex-A53 based embedded device.

Moreover, Figure 5 shows the trade-off of the running efficiency and the accuracy among different methods on Raspberry Pi 3b+. It can be seen that EagleEye outperforms the other methods on both speed and accuracy.

### 4.4. Memory Complexity

As mentioned in the introduction of this paper, the memory complexity of the algorithm has to be low enough to run on the embedded devices. Table 7 compares the EagleEye with other methods on the number of parameters, the model size, and the memory footprint. It can be concluded that the EagleEye achieved the lowest memory complexity among these methods. Moreover, EagleEye’s low memory complexity made it possible for EagleEye to run on almost all common embedded devices, for example the Raspberry Pi 3b+ (with the memory size of 1 GB) and the recent mobile phones (with memory size of >3 GB).

### 4.5. Comparison with State-of-the-Art on Wider Face Dataset

**Figure 6:**
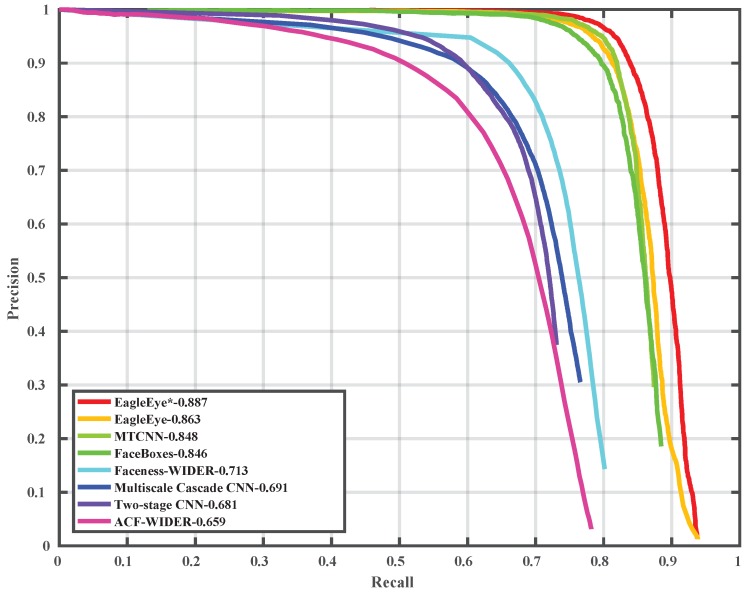
Precision-recall curve on wider face validation (easy) set.

**Figure 7:**
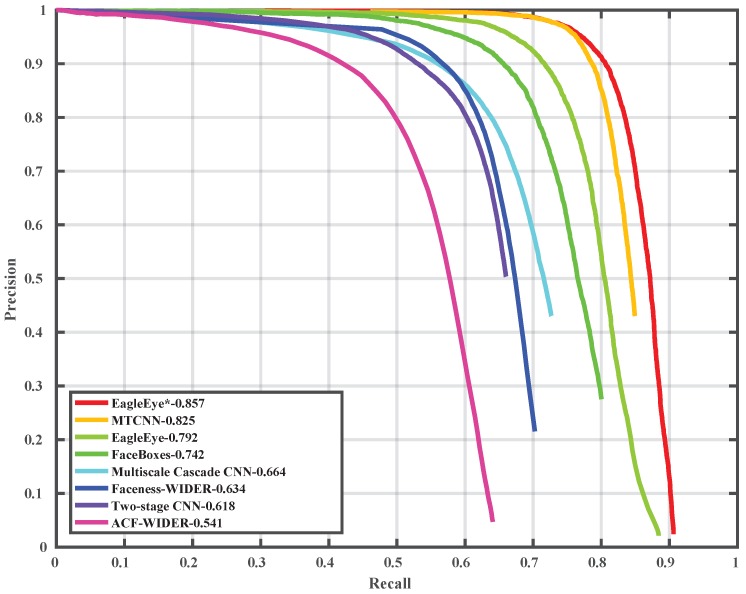
Precision-recall curve on wider face validation (medium) set.

**Figure 8:**
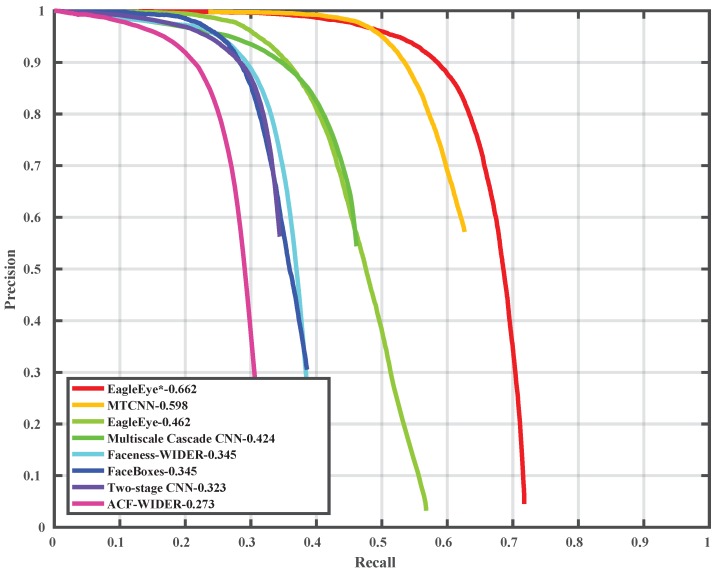
Precision-recall curve on wider face validation (hard) set.

**Figure 9:**
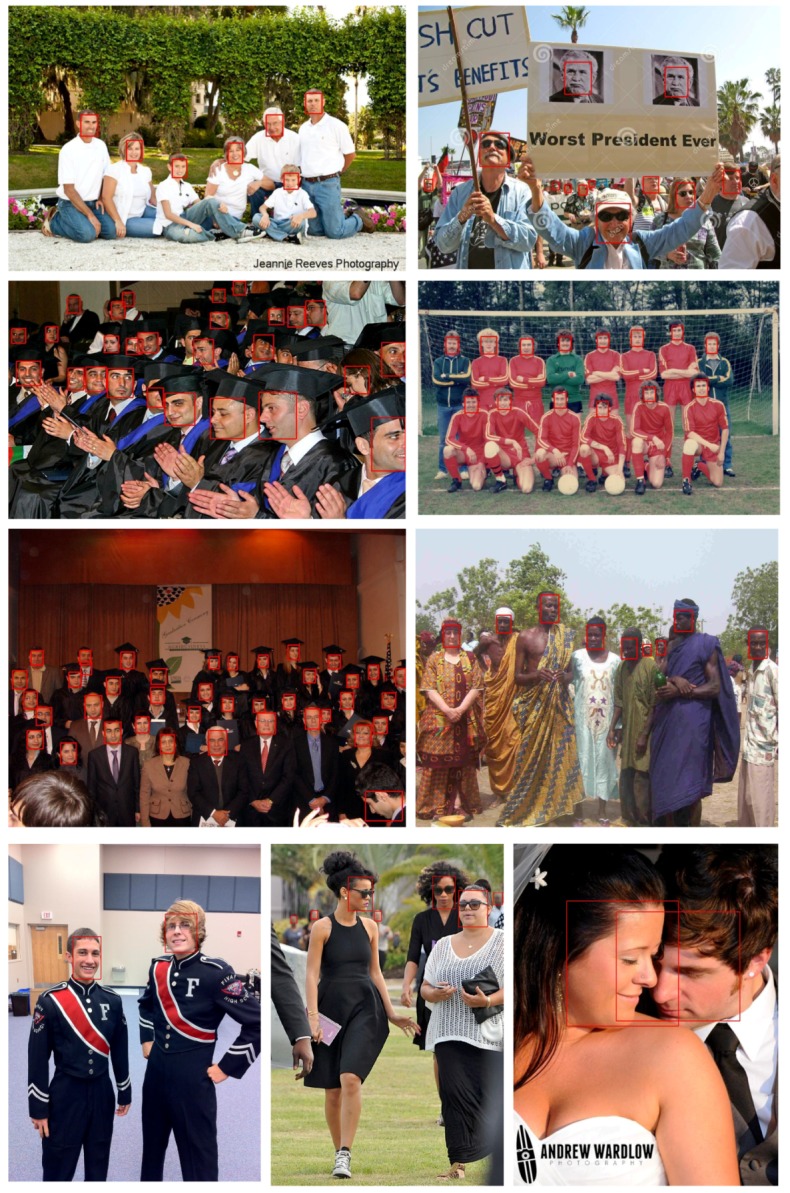
Visualization of the results of EagleEye on wider face dataset.

To compare the EagleEye’s performance with other state-of-the-art methods, we trained it with 1024 × 1024 image patches, instead of 512 × 512 image patches as in Section 4.2, to have a better performance. The precision-recall curves and mAP values are shown in Figure 6, Figure 7 and Figure 8. Since some detectors like MTCNN [24] use the image pyramid as input for better dealing with multi-scale faces, we also report the results of EagleEye by using multi-scale testing (image pyramid). In Figure 6, Figure 7 and Figure 8, “EagleEye” refers to the single-scale testing result of EagleEye, and “EagleEye*” refers to the multi-scale testing result. Generally, the EagleEye was better than most of the other methods [17,24,25,47,49] on three subsets, including Faceboxes [25], and it was better than all methods on the easy subset. After adopting multi-scale testing like MTCNN, EagleEye was able to outperform all methods on all three subsets. This experiment shows that besides the high runtime efficiency, EagleEye is also good in accuracy. Figure 9 shows some qualitative results on the wider face dataset.

### 4.6. Comparison with State-of-the-Arts on FDDB Dataset

**Figure 10:**
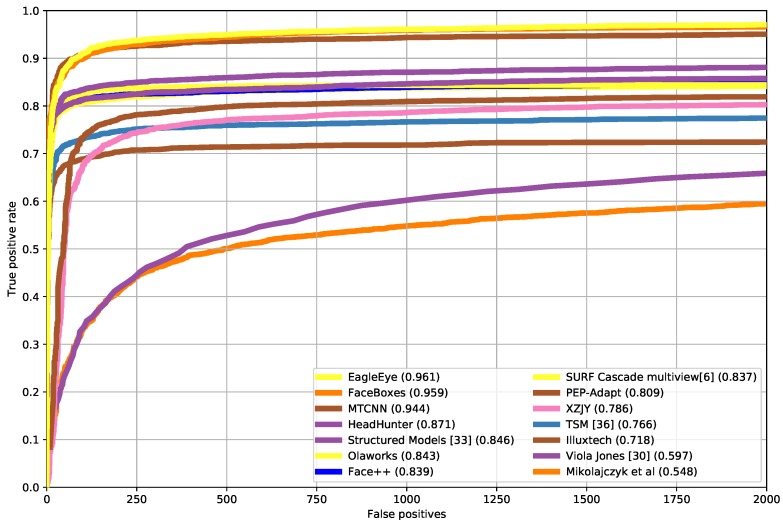
Discontinuous receiver operating characteristic (ROC) curves on the FDDB dataset.

**Figure 11:**
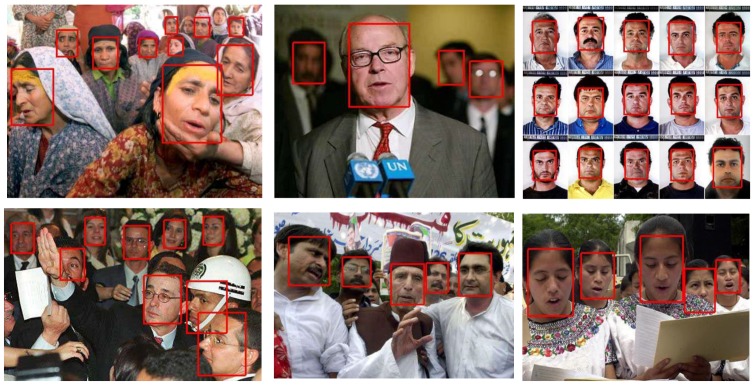
Visualization of the results of EagleEye on FDDB dataset.

The FDDB [48] dataset has 2845 images for testing the face detectors. It has 5171 annotated faces. We directly tested the detector trained on wider face in Section 4.5 on FDDB dataset and compared it with other state-of-the-art methods [24,25,49,50,51,52,53,54,55,56,57,58,59,60,61,62,63]. Figure 10 gives the discontinuous receiver operating characteristic (ROC) curve of the evaluation results. The performance of FDDB shown in Figure 10 is the true positive rate at 1000 false positives. It shows that EagleEye achieved the new state-of-the-art. Figure 11 shows some qualitative results on the FDDB dataset.

### 4.7. Comparison with State-of-the-Arts on PASCAL Face Dataset

**Figure 12:**
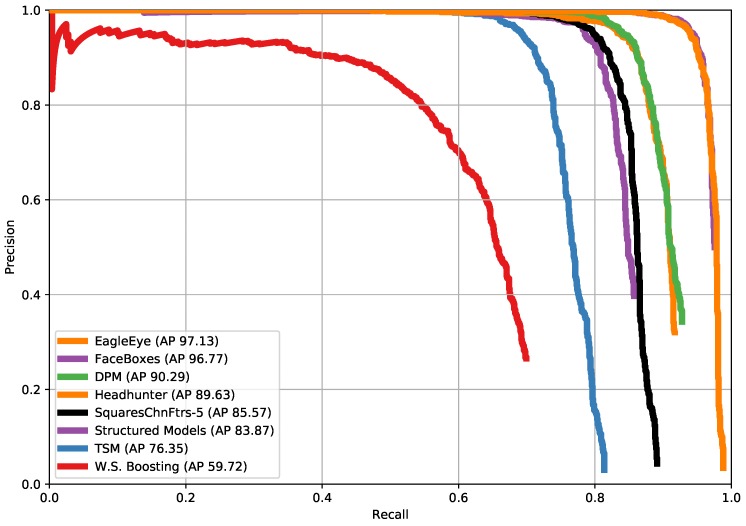
Precision-recall curves on PASCAL face dataset.

**Figure 13:**
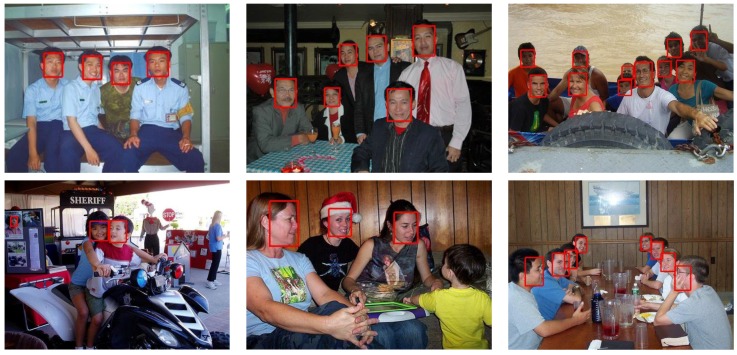
Visualization of the results of EagleEye on Pascal face dataset.

PASCAL face dataset consists of 851 images with 1335 faces. It is collected from the test set of PASCAL person layout dataset. The faces have large variations in appearances and poses. Like on FDDB, we tested the wider face trained detector on this dataset and compared it with other methods [25,49,64,65,66,67,68]. Figure 12 shows EagleEye also outperforms others at PASCAL face dataset. Figure 13 shows some qualitative results on the PASCAL face dataset.

## 5. Conclusions

In this paper, we propose a new face detector, EagleEye, which is specially designed for embedded devices. We propose five strategies for building it. The first two strategies are used to build efficient face detection networks that have low computation cost with enough capacity. We use convolution factorization to build the network with sparse connections and we use successive downsampling convolutions to remove the unnecessary layers in the first several stages. The latter three strategies are designed to improve the detection accuracy without affecting the FLOPS too much. We design the context module and insert it in the backbone network to extract context information for better detection and we choose the information preserving activation function, i.e., PReLU, to increase the capacity of the face detection network. We further use focal loss to get better performance by handling the class imbalance problem. With the five strategies, the EagleEye runs on the ARM Cortex-A53 based embedded device (Raspberry Pi1 3b+) at 21FPS with the input of VGA resolution with the better precision than the methods with the same order of computation complexity.

## Figures and Tables

**Table 1:** Architecture of the backbone of the baseline face detector.

Type/Stride	Filter Shape	Anchor Size
Conv/s2	3×3×3×4	—
Conv/s2	3×3×4×16	—
Conv/s2	3×3×16×32	—
Conv/s1	3×3×32×32	—
Conv/s2	3×3×32×64	—
4× Conv/s1	3×3×64×64	32, 32$\sqrt{2}$
Conv/s2	3×3×64×128	—
Conv/s1	3×3×128×128	64, 64$\sqrt{2}$
Conv/s1	1×1×128×96	—
Conv/s2	1×1×96×192	128, 128$\sqrt{2}$
Conv/s1	1×1×192×96	—
Conv/s2	3×3×96×192	256, 256$\sqrt{2}$

**Table 2:** Architecture of the backbone of EagleEye.

Type/Stride		Filter Shape	Anchor Size
Conv/s2		3×3×3×4	—
Conv dw/s2		3×3×4 dw	—
Conv/s1		1×1×4×16	—
Conv dw/s2		3×3×16 dw	—
Conv/s1		1×1×16×32	—
Conv dw/s1		3×3×32 dw	—
Conv/s1		1×1×32×32	—
Conv dw/s2		3×3×32 dw	—
Conv/s1		1×1×32×64	—
4×	Conv dw/s1	3×3×64 dw	—
	Conv/s1	1×1×64	—
Slice		—	—
Conv dw/s1/d1,		1×1×32 dw	—
Conv dw/s1/d2		1×1×32 dw	—
Concat		—	—
Conv/s1		1×1×64×64	32, 32$\sqrt{2}$
Conv dw/s2		3×3×64 dw	—
Conv/s1		1×1×64×128	—
Conv dw/s1		3×3×128 dw	—
Conv/s1		1×1×128×128	64, 64$\sqrt{2}$
Conv/s1		1×1×128×96	—
Conv dw/s2		1×1×96 dw	—
Conv/s1		1×1×96×192	128, 128$\sqrt{2}$
Conv/s1		1×1×192×96	—
Conv dw/s2		3×3×96 dw	—
Conv/s1		1×1×96×192	256, 256$\sqrt{2}$

**Table 3:** Ablation study on wider face’s validation set.

Contributions	Baseline					EagleEye512
Convolution Factorization		√	√	√	√	√
Successive Downsampling Convolutions			√	√	√	√
Context Module				√	√	√
Information Preserving Activation Function					√	√
Focal Loss						√
Accuracy (mAP[easy])	87.9	83.7	82.2	82.9	83.3	84.1
FLOPS	440.3 M	87.5 M	72.6 M	78.7 M	75.3 M	75.3 M

**Table 4:** Comparisons between different activation functions.

Method	mAP [Easy]	mAP [Medium]	mAP [Hard]	FLOPS
ReLU	82.9	76.5	46.5	72.6 M
PReLU	83.3	77.1	49.5	75.3 M
Leaky ReLU	83.4	76.8	48.2	75.3 M

**Table 5:** Comparison EagleEye with directly pruning on the baseline on wider face’s validation set.

Method	mAP [Easy]	mAP [Medium]	mAP [Hard]	FLOPS
Baseline	87.9	84.0	61.4	440.3 M
$\frac{1}{4}$ Baseline	74.7	65.5	34.7	80.7 M
EagleEye512	84.1	79.1	46.2	75.3 M

**Table 6:** Speed comparison with other face detection methods on FDDB with VGA input (640 × 480).

Method	mAP on FDDB	Desktop		ARM Based Embedded Devices	
		FPS	CPU (Desktop Devices)	FPS	CPU (Embedded)
ACF [17]	85.2	20	i7-3770@3.40	N/A	ARM Cortex-A53@1.4GHz
MTCNN [24]	94.4	16	N/A@2.60	5.4	ARM Cortex-A53@1.4GHz
Faceboxes [25]	96.0	20	E5-2660v3@2.60	3.4	ARM Cortex-A53@1.4GHz
$\frac{1}{8}$-SSD-MobileNet	96.0	20	E5-2660v3@2.60	10	ARM Cortex-A53@1.4GHz
EagleEye	96.1	21	E5-2660v3@2.60	20	ARM Cortex-A53@1.4GHz

**Table 7:** Memory complexity comparisons between different methods with VGA input.

Method	Parameters	Model Size	Memory Footprint
MTCNN [24]	0.50 M	1.90 MB	33.4 MB
Faceboxes [25]	0.91 M	3.87 MB	24.0 MB
$\frac{1}{8}$-SSD-MobileNet	0.93 M	3.59 MB	32.5 MB
EagleEye	0.23 M	0.952 MB	13.9 MB
